# Real-Time Estimation of Numerical Rating Scale (NRS) Scores Using Machine Learning-Based Facial Expression Analysis: A Proof-of-Concept Study

**DOI:** 10.7759/cureus.107883

**Published:** 2026-04-28

**Authors:** Kentaro Uejima, Tsutomu Takahashi, Miki Matsui, Sakae Fukushima, Yoshifumi Nishi

**Affiliations:** 1 School of Pharmacy, Nihon University, Chiba, JPN; 2 Department of Anesthesiology, Nihon University School of Medicine, Tokyo, JPN; 3 Department of Pharmacy, Nihon University Itabashi Hospital, Itabashi, JPN; 4 Center for Pharmacist Education, Nihon University School of Pharmacy, Funabashi, JPN

**Keywords:** computer vision, facial expression analysis, machine learning, non-verbal communication, numerical rating scale, pain assessment, palliative care, proof-of-concept

## Abstract

Background

Accurate pain assessment is fundamental to effective cancer pain management. However, subjective scales such as the Numerical Rating Scale (NRS) have limitations in pediatric and elderly patients with impaired verbal communication. This study aimed to develop a real-time facial expression-based pipeline for NRS estimation and to evaluate its technical feasibility as a proof of concept (PoC).

Methods

A real-time analysis pipeline was implemented in Python (version 3.11; Python Software Foundation, Fredericksburg, VA, USA), integrating MediaPipe for facial detection and DeepFace for emotion estimation. Seven emotion probability scores extracted from 30 fps video streams were used as predictor variables to estimate NRS values using several regression models, including Random Forest (RF). Synthetic datasets generated for technical validation were evaluated using leave-one-out cross-validation (LOOCV). Performance was assessed using Spearman’s rank correlation coefficient (ρ) and mean absolute error (MAE).

Results

In the pediatric dataset, the RF model achieved ρ = 0.7383 (p < 0.001) and MAE = 1.5195, demonstrating improved performance compared with the baseline model (ρ = 0.2765). In the elderly dataset, the RF model showed ρ = 0.7566 (p < 0.001) and MAE = 1.5760. Feature importance analysis indicated that “Fear” contributed prominently in both datasets, whereas “Neutral” also showed relatively higher importance in the elderly dataset.

Conclusions

This study demonstrated the technical feasibility of a real-time NRS estimation pipeline using artificial intelligence (AI)-based facial expression analysis. The findings suggest potential applicability as a complementary pain assessment approach for patients with limited verbal communication, pending future clinical validation.

## Introduction

Accurate pain assessment is fundamental to effective cancer pain management [1-3]. Subjective pain assessment scales such as the Visual Analogue Scale (VAS), Verbal Rating Scale (VRS), and Numerical Rating Scale (NRS) are widely used in patients capable of verbal communication [4,5]. However, adequate pain assessment may be challenging in certain populations, including pediatric and elderly patients with limited verbal communication abilities [6,7].

In this context, the Faces Pain Scale (FPS) is often employed as an alternative assessment tool. Pain-related facial expressions can be quantified using the Facial Action Coding System (FACS), which identifies specific action units (AUs) associated with pain intensity [8-10]. Nevertheless, because the FPS relies on subjective interpretation by patients or evaluators, its objectivity and reproducibility remain subject to observer variability despite reported inter-rater reliability [11-13]. Therefore, more objective and reproducible approaches for evaluating non-verbal pain information are warranted. Several observational tools, such as the Face, Legs, Activity, Cry, Consolability (FLACC) scale and Pain Assessment in Advanced Dementia (PAINAD), are widely used for pain assessment in non-verbal patients [14,15]. However, these approaches typically rely on manual and intermittent observation by healthcare providers. Our artificial intelligence (AI)-based approach aims to complement these existing tools by enabling more continuous and automated pain monitoring.

In this study, we conducted a technical proof-of-concept (PoC) investigation of a real-time facial expression analysis pipeline implemented in Python (Python Software Foundation, Fredericksburg, VA, USA) in a non-clinical setting. Although convolutional neural network (CNN)-based approaches for pain detection have been reported [16-19], studies focusing on real-time implementation and its quantitative association with NRS-based estimation remain limited. Therefore, as an initial step toward developing a clinically applicable system, this study aimed to investigate the technical feasibility of real-time NRS estimation using a controlled synthetic dataset, allowing reproducible validation of the proposed analysis pipeline without ethical constraints related to patient facial data.

## Materials and methods

Study design

This study was conducted as a PoC investigation to verify the technical feasibility of a real-time pain estimation system based on facial expression analysis, with potential future applications in cancer pain management. The primary objective was to establish an analysis pipeline and evaluate algorithmic performance in a controlled, non-clinical setting. This study was not intended to provide clinical validation.

Research environment and platform

The analysis pipeline was implemented in a JupyterLab environment using Python (version 3.11). The primary libraries utilized were as follows: NumPy and Pandas (for numerical computation and data processing), OpenCV (cv2) (for image acquisition and pre-processing), MediaPipe (for facial region detection and tracking), DeepFace (for emotion probability estimation), and scikit-learn (for regression model development and evaluation). To enhance reproducibility, the random_state parameter was fixed in all regression models to eliminate the influence of stochastic variations.

Facial analysis and feature extraction

Static facial images were processed through a real-time analysis pipeline operating at 30 frames per second (fps) to simulate continuous video input. Each frame was converted to RGB format using OpenCV, followed by face detection and tracking via MediaPipe.

Detected facial regions were analyzed using the DeepFace framework, which utilizes pre-trained deep learning models to estimate emotion probabilities. For each frame, probabilities (ranging from 0 to 1) were generated for seven emotion categories: happy, surprise, neutral, sad, angry, fear, and disgust. These seven probabilities were extracted as independent variables (features) for subsequent regression modeling. Frames in which face detection failed were excluded from the analysis.

Validation data

For technical verification, facial images generated using AI were utilized as validation data. Images representing pediatric and elderly facial models were generated, with each image assigned a target NRS value ranging from 1 to 10 at the time of generation. The NRS, a widely used 0-10 pain assessment scale, was used to evaluate pain intensity [4]. As the NRS is a non-proprietary scale widely used in clinical practice and research, explicit copyright permission was not required for its application in this study. To ensure methodological transparency and reproducibility, facial images for each NRS level were generated using the Fotor AI Image Generator (Fotor, Inc., Sichuan, China, accessed in March 2026). Representative prompts included specific descriptors of age, gender, and pain intensity, such as “A close-up photo of an elderly person's face expressing severe agonizing pain, corresponding to NRS 8” or “A face of a child showing mild discomfort, NRS 2.” Each generated image was initially assigned a target NRS value based on pre-defined prompts during the generation process. Subsequently, all images underwent post hoc visual inspection by an experienced researcher (a pharmacist specializing in palliative care) to confirm that the facial expressions clinically aligned with the intended NRS scores. Images that did not sufficiently match these criteria were excluded to ensure label consistency and reliability. This process was implemented to enhance both the consistency and reproducibility of the assigned NRS labels. A total of 60 images (pediatric: n = 30, 50.0%; elderly: n = 30, 50.0%) were used, with multiple images corresponding to each NRS value. These images did not involve real human subjects. The assigned NRS values served as reference target labels for algorithm evaluation. Importantly, the assigned NRS values do not represent clinically validated pain intensity; rather, they were used to assess algorithmic behavior under controlled synthetic conditions.

Development and selection of the NRS estimation model

Using the seven extracted emotion probabilities as independent variables and the pre-set NRS values as target variables, the following regression models were evaluated: Random Forest (RF) Regressor, Gradient Boosting Regressor, Ridge Regression, and Lasso Regression. For linear models (Ridge and Lasso), feature standardization using Z-score normalization was performed within each fold during the validation process to prevent data leakage. Given the limited dataset size, leave-one-out cross-validation (LOOCV) was employed to assess generalization performance.

Evaluation metrics

Model performance was assessed using the following metrics.

Spearman’s Rank Correlation Coefficient (ρ)

Spearman’s ρ was calculated to evaluate the monotonic association between estimated and reference NRS values.

Mean Absolute Error

Mean absolute error (MAE) was calculated as

$\mathrm{MAE} = \frac{1}{n} \sum_{i=1}^{n} |y_i - \hat{y}_i|$

where y_i_ represents the reference NRS and ŷ_i_ represents the estimated NRS score.

The model demonstrating the highest ρ and lowest MAE was considered to have the most stable technical estimation performance.

Validation of the real-time analysis pipeline

To evaluate real-time functionality, a one-minute continuous frame analysis was performed for each static image processed through the 30 fps simulation pipeline. The median of the frame-by-frame NRS estimates was defined as the representative value for each image to reduce the influence of transient prediction fluctuations across frames. Estimated NRS values were displayed in real time on the monitor to assess processing latency and system stability.

Ethical considerations

This study was conducted in a non-clinical setting using AI-generated images and did not involve real human subjects. The Ethics Committee of the Nihon University School of Pharmacy determined that the study was exempt from ethical review.

## Results

Overall estimation performance

**Figure 1:**
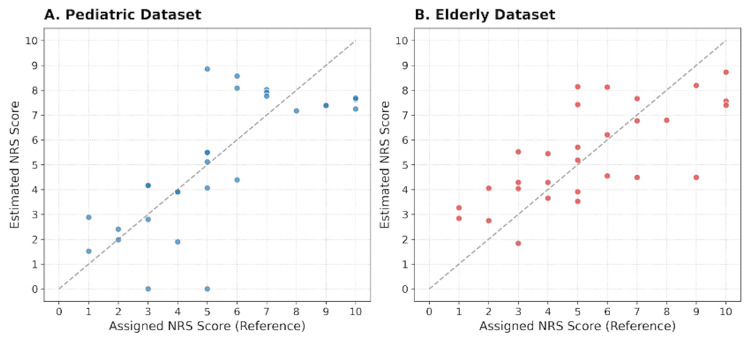
(A, B) Relationship between reference and estimated NRS scores in pediatric and elderly synthetic datasets. The dashed line indicates the identity line (y = x). Statistical correlation was assessed using Spearman’s rank correlation coefficient (ρ). Statistical significance was defined as p < 0.05. Data are presented as mean ± standard deviation (SD). NRS: Numerical Rating Scale

The proposed analysis pipeline demonstrated moderate-to-strong positive correlations between the reference NRS values and the estimated scores in both the pediatric and elderly datasets (Figure 1).

Scatter plots show the relationship between reference NRS labels and estimated NRS scores produced by the proposed pipeline in pediatric and elderly synthetic datasets. In the pediatric dataset, the RF model achieved Spearman’s rank correlation coefficient of ρ = 0.7383 (p = 4.84 × 10⁻⁶) with a MAE of 1.5195. In the elderly dataset, the same model showed ρ = 0.7566 (p = 1.31 × 10⁻⁶) with an MAE of 1.5760.

Model comparison in the pediatric dataset

**Figure 2:**
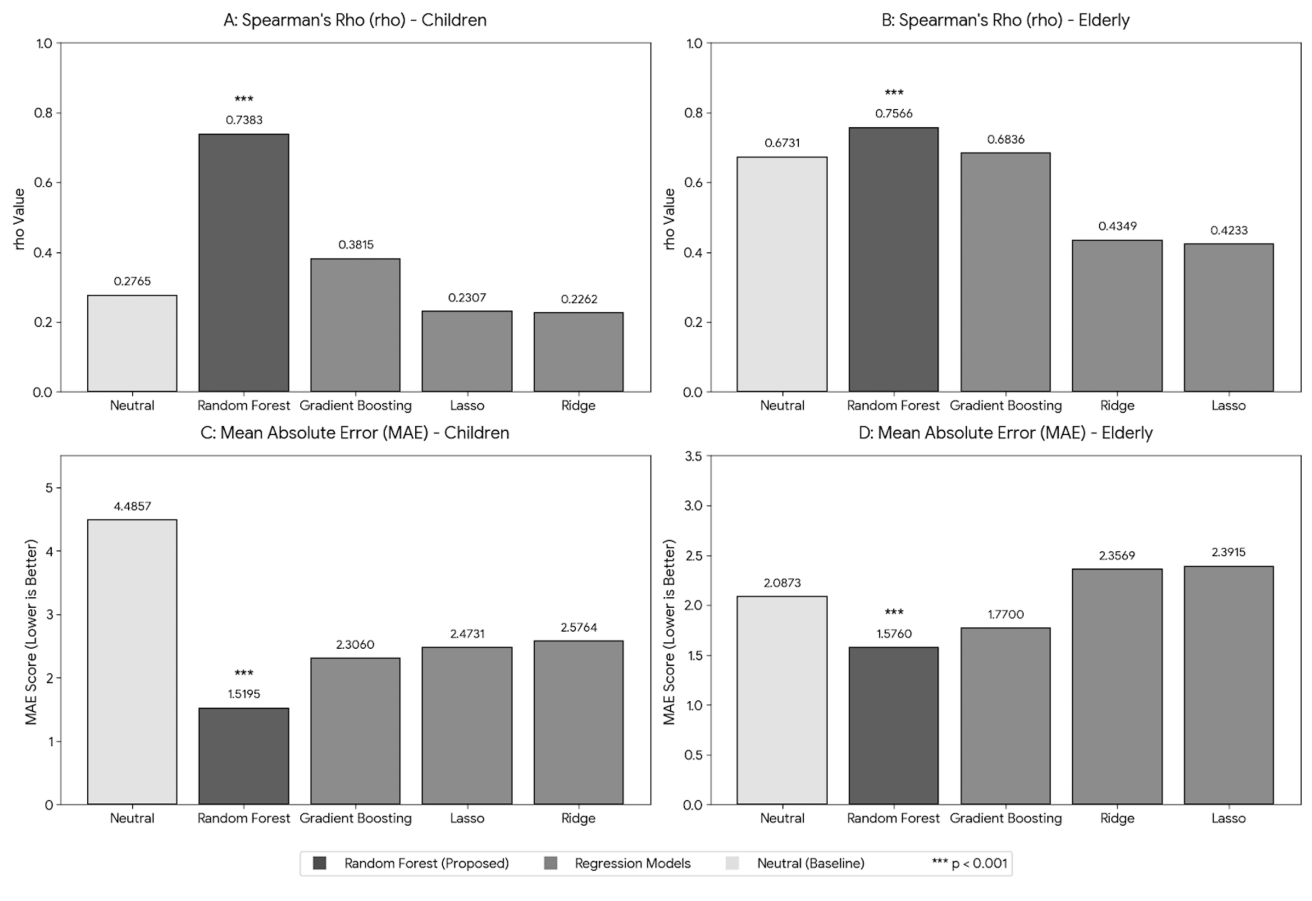
Comparison of regression model performance for NRS estimation in pediatric and elderly synthetic datasets. Panels show Spearman’s rank correlation coefficients (ρ) and mean absolute error (MAE) across regression models. Statistical comparisons between models were performed using the Wilcoxon signed-rank test, and significance was defined as p < 0.05. *** denotes p < 0.001. Data are presented as mean ± standard deviation (SD). (A) Spearman’s ρ in the pediatric dataset. (B) Spearman’s ρ in the elderly dataset. (C) MAE in the pediatric dataset. (D) MAE in the elderly dataset. The baseline model used only the “Neutral” emotion probability as a predictor. The Random Forest (proposed) model demonstrated the highest ρ and the lowest MAE in both groups. NRS: Numerical Rating Scale

Within the pediatric dataset, non-linear models demonstrated moderate-to-strong correlations between the estimated and reference NRS values. Specifically, non-linear models (RF and Gradient Boosting) consistently exhibited higher correlation coefficients and lower MAE values compared with linear models (Ridge and Lasso Regression) (Figure 2).

These findings suggest that non-linear approaches may better accommodate the variability observed in facial features within the present pediatric dataset.

Comparison with the baseline model in the elderly dataset

In the elderly dataset, the performance of the RF model was compared with a baseline model constructed using only the Neutral emotion score. The RF model demonstrated a higher correlation coefficient (increase of 0.0835) and a lower MAE (reduction of 0.5112) compared with the baseline. These findings suggest improved estimation performance when multiple emotional features were incorporated, within the constraints of the present experimental setting.

Feature Importance Analysis

**Figure 3:**
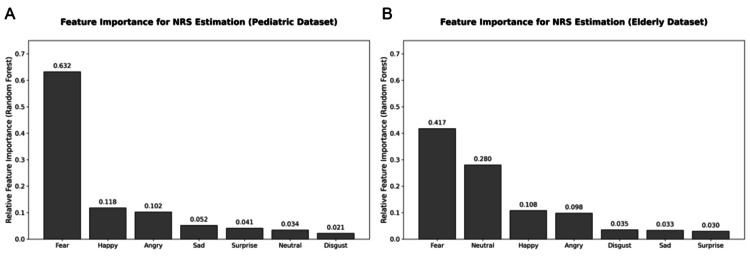
(A, B) Feature importance of emotional probabilities for NRS estimation using Random Forest. Bars indicate relative contributions of emotion probabilities to the estimated NRS score in pediatric and elderly synthetic datasets. Data represent feature importance scores derived from the Random Forest model. These trends reflect patterns observed in the synthetic validation dataset and do not imply direct physiological interpretations. NRS: Numerical Rating Scale

Feature importance analysis of the RF model in the elderly dataset showed that Fear, Neutral, and Happy had relatively higher importance scores compared with other features (Figure 3).

These findings suggest that, within this synthetic dataset, the probabilities of these emotional categories were more strongly associated with the reference NRS values.

## Discussion

Technical feasibility of the AI-based pain estimation model

In this study, a moderate-to-strong positive correlation between reference NRS values and estimated scores was observed in both pediatric and elderly models using RF. Notably, in the pediatric dataset, the RF model demonstrated improved correlation compared to the baseline model. These findings suggest that non-linear models capable of integrating multiple emotional probabilities may provide more stable estimation performance than simple linear approaches within the present experimental framework. The theoretical foundation for using multiple emotional features to estimate NRS scores lies in the conceptualization of pain as a multidimensional experience. The International Association for the Study of Pain (IASP) defines pain not only as a sensory stimulus but also as “an unpleasant sensory and emotional experience” [20]. Previous studies have demonstrated that pain-related facial expressions are composed of a complex blend of negative affective components, including fear, disgust, and sadness [10]. Therefore, the integration of multiple emotional probabilities may reflect the emotional complexity underlying pain perception. By employing a multivariate analysis of these features, our model attempts to capture the multidimensional nature of pain more comprehensively than simple linear approaches. Although this study represents a technical PoC and does not establish clinical validity, the successful implementation of a real-time NRS estimation pipeline using facial expression analysis represents an important technical step toward future clinical applications.

Age-specific feature trends

Feature importance analysis revealed distinct trends in emotional variables contributing to NRS estimation between the pediatric and elderly datasets. In the pediatric dataset, “Fear” showed a relatively high contribution, whereas in the elderly dataset, both “Fear” and “Neutral” demonstrated comparatively higher importance scores.

It is important to emphasize that these observations reflect patterns specific to the present synthetic dataset and do not provide direct physiological explanations of pain expression mechanisms. Validation using clinical video data from real patients will be necessary to determine the clinical relevance of these emotional features.

Technical validity of algorithm selection

Comparison of multiple regression models indicated that non-linear approaches such as RF outperformed linear models under the current conditions. This suggests that the relationship between facial features and NRS intensity may exhibit non-linear characteristics within this dataset. Additionally, RF demonstrated a favorable balance between estimation stability and computational efficiency, supporting its suitability for real-time analysis scenarios where low latency is required.

In terms of computational performance, the system achieved a processing speed of 30 fps. This high-speed processing supports the model’s low computational latency and temporal stability, which are critical for real-time clinical monitoring. Moreover, the calculation of median NRS values over a one-minute analysis window was intentionally designed as a noise-reduction strategy. By utilizing the median rather than a simple mean, the model reduces the impact of transient outliers, such as momentary facial artifacts or detection errors, thereby providing more robust and stable pain intensity estimation in real-time settings.

Clinical implications and future prospects

An advantage of the proposed system is its ability to operate in standard computing environments using conventional camera devices, without requiring specialized sensors. This accessibility may allow the technology to serve as a potential framework for continuous pain monitoring in non-verbal patients, pending further clinical validation.

Future studies should include observational investigations using video data from actual cancer patients to examine correlations with self-reported NRS values and responsiveness to analgesic interventions. It should be noted that the present findings are based on synthetic AI-generated images and therefore may not directly reflect real-world clinical pain expression. Future research should aim to transition from static image simulation to continuous video analysis to capture the temporal dynamics of pain expression. Furthermore, incorporating pain-specific facial AUs in addition to basic emotion probabilities could improve the model’s sensitivity to subtle pain cues. Validating the system with larger real-world datasets and testing its robustness under realistic clinical constraints-such as poor lighting, suboptimal camera angles, and facial occlusion-will be essential to enhance its clinical relevance and ensure its reliability in diverse healthcare settings.

Limitations

Several limitations should be acknowledged. First, the dataset size was relatively small. Second, the study relied on synthetic images generated under controlled conditions, which may not fully capture the variability in lighting, posture, and individual facial expression differences encountered in clinical environments.

Furthermore, it is important to acknowledge that AI-generated facial expressions may contain model-specific artifacts, which represent an inherent limitation of this PoC study. Therefore, the assigned NRS values in this dataset are best interpreted as logical benchmarks for algorithm verification rather than absolute clinical truths. This controlled synthetic framework was specifically designed to evaluate the technical performance and stability of the estimation pipeline prior to future clinical validation using real patient data.

Moreover, the assigned NRS values in the synthetic dataset do not represent clinically validated pain intensity ratings but were used to evaluate algorithmic behavior. External validation using independent datasets was not performed. Additionally, pediatric image generation may have been influenced by platform-specific constraints, potentially limiting expression diversity.

An additional limitation of this study is that the model utilized basic emotion probabilities rather than pain-specific facial AUs based on the FACS. While basic emotional features may reflect the multidimensional nature of pain, they may not fully capture pain-specific facial expressions or adequately distinguish pain from other negative emotional states. Future investigations should incorporate real patient data and FACS-based AU detection to improve the robustness, specificity, and clinical relevance of the proposed estimation approach.

## Conclusions

This PoC study demonstrated the technical feasibility of a real-time pain estimation pipeline using machine learning-based facial expression analysis. By integrating multiple emotional probabilities through an RF Regressor, the system achieved moderate-to-strong correlations with reference NRS values in both pediatric and elderly synthetic datasets, showing improved performance compared with baseline evaluations.

The proposed framework, which operates in standard computing environments without the need for specialized sensors, may serve as a foundational technical framework for future investigation of objective pain monitoring approaches in non-verbal or vulnerable patient populations. Further validation using clinical data from real patients is required to assess robustness, generalizability, and clinical utility. These findings represent an initial step toward improving objectivity in pain assessment methodologies within diverse clinical settings.
